# Prolonged P300 Latency in Antipsychotic-Free Subjects with At-Risk Mental States Who Later Developed Schizophrenia

**DOI:** 10.3390/jpm11050327

**Published:** 2021-04-21

**Authors:** Yuko Higuchi, Tomiki Sumiyoshi, Takahiro Tateno, Suguru Nakajima, Daiki Sasabayashi, Shimako Nishiyama, Yuko Mizukami, Tsutomu Takahashi, Michio Suzuki

**Affiliations:** 1 Department of Neuropsychiatry, Graduate School of Medicine and Pharmaceutical Sciences, University of Toyama, Toyama 930-0194, Japan; tdtpodim@med.u-toyama.ac.jp (T.T.); snaka@med.u-toyama.ac.jp (S.N.); ds179@med.u-toyama.ac.jp (D.S.); nishiyas@ctg.u-toyama.ac.jp (S.N.); yk1022@med.u-toyama.ac.jp (Y.M.); tsutomu@med.u-toyama.ac.jp (T.T.); suzukim@med.u-toyama.ac.jp (M.S.); 2 Research Center for Idling Brain Science, University of Toyama, Toyama 930-0194, Japan; 3 National Center of Neurology and Psychiatry, Department of Preventive Intervention for Psychiatric Disorders, National Institute of Mental Health, Tokyo 187-8551, Japan; sumiyot@ncnp.go.jp; 4 Center for Health Care and Human Sciences, University of Toyama, Toyama 930-8555, Japan

**Keywords:** P300, event-related potentials, clinical high-risk, psychosis, at risk mental state, schizophrenia, cognition, latency

## Abstract

We measured P300, an event-related potential, in subjects with at-risk mental states (ARMS) and aimed to determine whether P300 parameter can predict progression to overt schizophrenia. Thirty-three subjects with ARMS, 39 with schizophrenia, and 28 healthy controls participated in the study. All subjects were antipsychotic-free. Subjects with ARMS were followed-up for more than two years. Cognitive function was measured by the Brief assessment of Cognition in Schizophrenia (BACS) and Schizophrenia Cognition Rating Scale (SCoRS), while the modified Global Assessment of Functioning (mGAF) was used to assess global function. Patients with schizophrenia showed smaller P300 amplitudes and prolonged latency at Pz compared to those of healthy controls and subjects with ARMS. During the follow-up period, eight out of 33 subjects with ARMS developed overt psychosis (ARMS-P) while 25 did not (ARMS-NP). P300 latency of ARMS-P was significantly longer than that of ARMS-NP. At baseline, ARMS-P elicited worse cognitive functions, as measured by the BACS and SCoRS compared to ARMS-NP. We also detected a significant relationship between P300 amplitudes and mGAF scores in ARMS subjects. Our results suggest the usefulness of prolonged P300 latency and cognitive impairment as a predictive marker of later development of schizophrenia in vulnerable individuals.

## 1. Introduction

Patients with schizophrenia suffer from impairments of several types of cognitive functions which are considered to affect quality of life (QOL) and social functions [1]. Therefore, early detection and intervention into cognitive disturbances of schizophrenia are needed to achieve a satisfactory outcome for patients. For the same reason, the im-portance of intervention into the prodromal stage of schizophrenia and other psychotic disorders has also been recognized [2,3,4]. For this purpose, operational criteria to detect putative prodromal symptoms of psychosis have been used worldwide [5], and are designated as ultra-high risk, clinical high risk, or at-risk mental state (ARMS). These criteria allow for the identification of subjects with ~30% risk of developing psychosis over a two-year period [6], mostly schizophrenia-spectrum disorders [7]. For better prediction, the use of objective biomarkers, such as those based on brain morphology, neurophysiology, and neuropsychology, has been proposed [8,9,10,11,12]. For example, event-related potentials (ERPs), such as duration mismatch negativity (MMN) and P300, have been reported to provide sensitive and feasible electrophysiological tools [13,14]. These measures of ERPs have also been suggested to provide biological substrates for some aspects of cognitive disturbances in patients with schizophrenia and ARMS subjects [9,15,16].

P300 was discovered in 1960s and has been shown to reflect a putative electrophysiological basis of cognitive functions, such as attention-dependent information processing and immediate memory [17,18,19], which are impaired in schizophrenia [20,21]. Specifically, patients with schizophrenia have been reported to elicit smaller amplitudes and pro-longed latencies of P300 compared to those in healthy control subjects [22,23,24]. In fact, a recent meta-analysis reported that the effect sizes of amplitude reduction and latency prolongation are as large as 0.83 and 0.48, respectively [25]. As in the case for schizophrenia, abnormalities in P300 have been reported in other neuropsychiatric disorders, such as mood disorders (e.g., bipolar disorder and major depression), developmental disorders (e.g., attention deficit hyperactivity disorder), and Parkinson’s disease [26,27,28,29,30,31]. Therefore, P300 may be considered to provide a biomarker of schizophrenia, especially in patients in whom these diseases are excluded.

Potential P300 abnormalities have also been examined in subjects with ARMS [32,33,34,35,36,37,38]. To our knowledge, five studies to date have attempted to determine whether P300 parameters (e.g., amplitude and latency) at baseline can predict later onset of overt psychosis [33,34,35,36,37]. Two studies [34,36], but not others [33,35,37] reported differences in amplitude between ARMS subjects who later developed psychosis (ARMS-P) and those who did not (ARMS-NP). On the other hand, these previous ARMS studies did not find associations between baseline P300 latency and later psychosis onset [33,35,37]. These negative results may contradict the concept that prolonged P300 latency would reflect trait abnormality of schizophrenia irrespective of illness stages, including the prodromal stage [25]. The reasons for these inconsistent findings in ARMS remain unclear but may include the difference in clinical profiles of participants, e.g., medication status that has been reported to alter P300 parameters [9,39,40]. Thus, further investigations to examine the potential ability of P300 latency to predict later onset of psychosis in high-risk subjects would be desired.

In this study, we reported P300 amplitudes and latencies in subjects with antipsychotics-free ARMS or schizophrenia in comparison with healthy control subjects. On the basis of P300 abnormalities as a trait marker of schizophrenia [25], we hypothesized that baseline P300 latency in ARMS subjects would predict onset of psychosis. We also explored whether abnormal P300 parameters would be associated with cognitive and functional deficits in these subjects.

## 2. Materials and Methods

### 2.1. Participants

Thirty-three ARMS subjects (male/female, 23/10; mean age 19.2 ± 4.6 years), recruited from University of Toyama Hospital or Toyama Prefectural Mental Health Centre [41], as well as 39 schizophrenia patients (male/female, 16/23; mean age 24.4 ± 7.2 years) participated in this study. Diagnosis was made by experienced psychiatrists based on ICD-10 for schizophrenia and the Comprehensive Assessment of At-Risk Mental State (CAARMS) for ARMS [42]. We also recruited 28 healthy volunteers (male/female, 16/12; mean age 21.7 ± 5.0 years) from our community. All subjects were physically healthy and had well hearing ability. A psychiatric and treatment history was collected from the subjects themselves, their families, and medical records. Exclusion criteria included the following: subjects with a history of substance abuse or dependence, seizure, and head injury. Eligible patients were confirmed to be physically healthy by physical examinations and standard laboratory tests. Healthy volunteers and their first-degree relatives did not have any psychiatric disorders. Subjects with an estimated premorbid IQ less than 70 were also excluded.

For the clinical assessments, experienced psychiatrists administered the Positive and Negative Syndrome Scale (PANSS) [43]. The Japanese adult reading test (JART) [44] was performed to estimate premorbid IQ. The Brief Assessment of Cognition in Schizophrenia (BACS) [45,46], Schizophrenia Cognition Rating Scale (SCoRS) [47,48,49] and modified Global Assessment of Functioning (mGAF) [50] were used to evaluate cognitive and social functions. BACS was standardized by z-scores, with the mean score of Japanese healthy controls set to zero and the standard deviation set to one [51]. Furthermore, the BACS composite score was calculated by averaging the z-scores of the six primary BACS measurements [45]. The demographic data at baseline evaluation for healthy control, ARMS and schizophrenia is shown in Table 1.

Subjects with ARMS were clinically followed-up at our hospital. When the severity of psychotic symptoms exceeded the criteria of CAARMS, the subject was regarded as ARMS-P. In this study, eight out of the 33 (24.2%) subjects with ARMS developed schizophrenia during the observation period (1.0 ± 1.1 years). Twenty-five subjects who did not develop psychosis were defined as ARMS-NP. The observation period for ARMS-NP was more than two years, with average 3.5 ± 2.3 years. The demographic data at baseline evaluation for ARMS-NP and ARMS-P is shown in Table 2.

No subject was on antipsychotic medications. All ARMS-NP patients (twenty-five), five of eight ARMS-P patients and thirty one out of thirty-nine schizophrenia patients were antipsychotic naïve. Other subjects were antipsychotic free at least 2 weeks.

This protocol was approved by the Committee on Medical Ethics of the University of Toyama. After complete and detail description of the study was provided, written informed consent was obtained from the participants in agreement with the Declaration of Helsinki. If a subject was under 20 years old, written consent was also obtained from a parent or legal guardian.

### 2.2. Electroencephalogram (EEG) Recording

ERPs were recorded at the time of clinical assessments using an auditory odd-ball paradigm, based on an established method [9,22,39]. Subjects were directed to lay awake on a bed, open their eyes, and watch a red circle shown on a display monitor. The patients were observed carefully, and if the patients were in poor conditions (asleep, too many eye blinks or eye movements, frequent body movement, unwilling to participate in the examination), we gave instructions again or stopped the recording.

EEGs were recorded with a 32-channel DC-amplifier (EEG-2100 version 2.22J, Nihon Kohden Corp., Tokyo, Japan). Recordings were performed using 19 or 32 channel Electrocap (Electrocap Inc., Eaton, OH) or in a wave-shielded and sound-attenuated room. Auditory stimuli were delivered binaurally through headphones with variable inter-stimulus intervals ranging from 1.5 to 2.5 s. Target tones of 2000 Hz were randomly presented in a series of standard tones of 1000 Hz, with the presentation probability of 0.2 for target tones. Numbers of the standard and target (deviant) tones were 200 and 50, respectively. All tones were 50 ms in duration with a rise–fall time of 10 ms. Subjects were requested to press a button as promptly and accurately as possible in response to infrequent target tones. EEGs were recorded at 19 (located at FP1, FP2, F3, F4, F7, F8, C3, C4, P3, P4, O1, O2, T3, T4, T5, T6, Fz, Cz, and Pz) or 29 (Fp1, Fp2, F3, F4, F7, F8, FC3, FC4, C3, C4, T3, T4, CP3, CP4, TP7, TP8, P3, P4, T5, T6, O1, O2, FPz, Fz, FCz, Cz, CPz, Pz, and Oz) electrodes with average reference. In this paper, only midline (Fz, Cz, and Pz) and temporal lobe (T3, and T4) electrodes were presented according to a previous study [9]. The bandwidth was 0.16–120 Hz with a 60 Hz notch filter. Electrode impedance was less than 10 kΩ. Data were collected with a sampling rate of 500 Hz. Averaging of ERP waves and related procedures were performed using EPLYZER II software (Kissei Comtec, Co. Ltd. Nagano, Japan). The epoch was 700 ms, including a 100 ms pre-stimulus baseline. EEG responses with target tones were averaged off-line. We averaged all pre-stimulus amplitudes (from −100 to 0 ms) and defined it as zero-point. The peak of P300 was observed 250–400 ms after the target sound started. We defined it as time-window, and a positive maximum peak was detected within this time window by EPLYZAR II software. P300 amplitude was defined as the difference between the zero-point and peak, and its latency was defined as the interval from 0 ms to the timing of the peak. Before averaging ERPs, we manually checked the raw waveforms with care and removed all epochs without a typical P300 shape due to eye movement or blinking, facial muscle movement, sweating, or basic rhythms.

### 2.3. Statistical Methods

We used Statistical Package for Social Sciences (SPSS) version 25 (SPSS Japan Inc., Tokyo, Japan) for statistical analyses. Groups were compared for demographic and clinical data using a one-way analysis of variance (ANOVA) or Chi-square test. We used analysis of covariance (ANCOVA) with group as a between-subject variable and age as a covariate to evaluate group differences in P300 amplitude and latency, SCoRS score and GAF score. BACS domains were corrected by age and gender, as reported previously [45]. Bonferroni’s tests were employed as post hoc analysis to follow-up results yielded by ANCOVA. Correlation analyses were carried out to study the relationship between the amplitude of P300 vs. performance on clinical and neuropsychological tasks by Spearman’s rank correlation test. In these correlational analyses, we used amplitudes at Pz as a representative parameter, according to previous studies [52,53].

For data from the six subtests on the BACS, Bonferroni’s tests were performed for multiple comparisons. In subjects with ARMS, P300 amplitude and latency were normally distributed, with no differences between ARMS-P and ARMS-NP demographics; therefore, they were compared by an independent sample *t*-test.

For all statistical analyses, Shapiro–Wilk analysis and Levene’s test were performed to test normality, and significance level was defined as *p* < 0.05.

## 3. Results

### 3.1. Profiles of Subjects

Demographic and clinical data of the participants with healthy controls, ARMS and schizophrenia is shown in Table 1. There was a significant group difference in age and PANSS positive symptom between ARMS and schizophrenia, while the male/female ratio and JART scores did not differ. mGAF scores in patients with schizophrenia were worse than those of subjects with ARMS. Scores on the composite score of the BACS was lowest for schizophrenia group, followed by ARMS, compared to healthy control group. As shown in Table 2, compared to ARMS-NP group, ARMS-P group was not different in gender, age, JART, PANSS, and mGAF, but ARMS-P had worse scores on the BACS and SCoRS at baseline.

### 3.2. Group Comparison of P300 in Healthy Control, ARMS, and Schizophrenia

**Figure 1:**
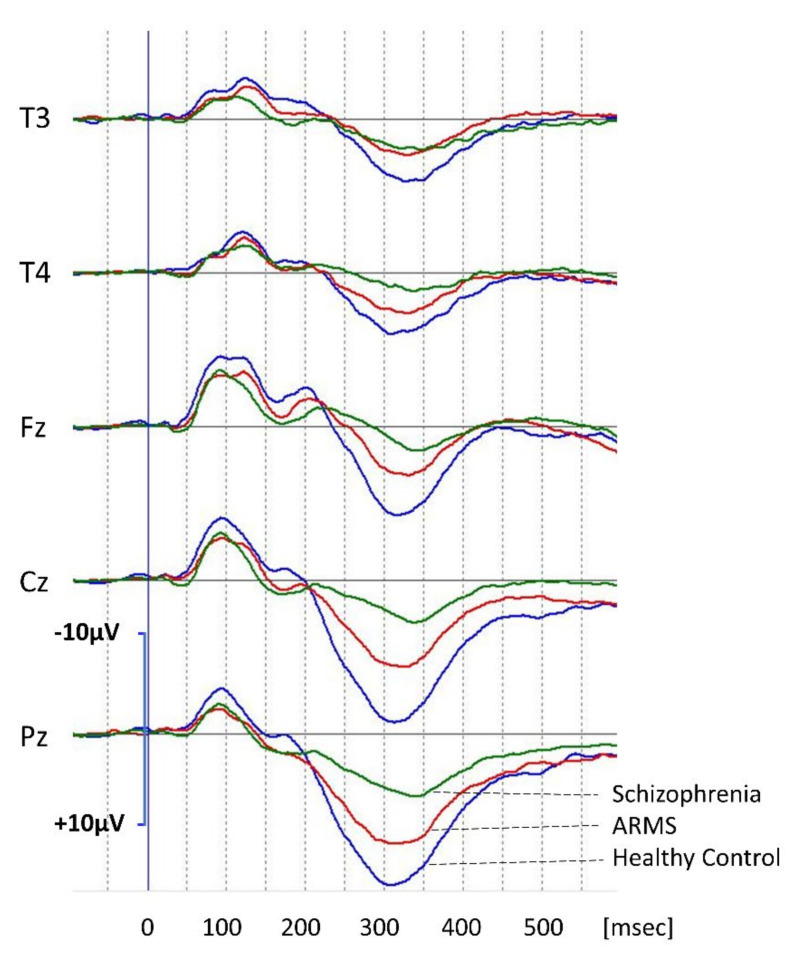
P300 waveforms at the T3, T4, Fz, Cz, and Pz leads. Grand average waveforms in healthy controls (Control, blue lines), subjects with at-risk mental states (ARMS, red lines), and patients with schizophrenia (Schizophrenia, green lines), respectively.

Grand average waveforms of P300 for healthy control, ARMS, and schizophrenia are shown in Figure 1. Average P300 amplitudes and latencies of these three groups at the T3, T4, Fz, Cz, and Pz leads are shown in Table 1. Statistical analysis revealed that patients with schizophrenia showed smallest P300 amplitudes, followed by ARMS and healthy control, at midline electrodes. P300 latencies at Cz and Pz in schizophrenia were longer than those in healthy controls and ARMS. P300 latency in subjects with ARMS do not differ from those in healthy control.

### 3.3. P300 between ARMS-P and ARMS-NP

**Figure 2:**
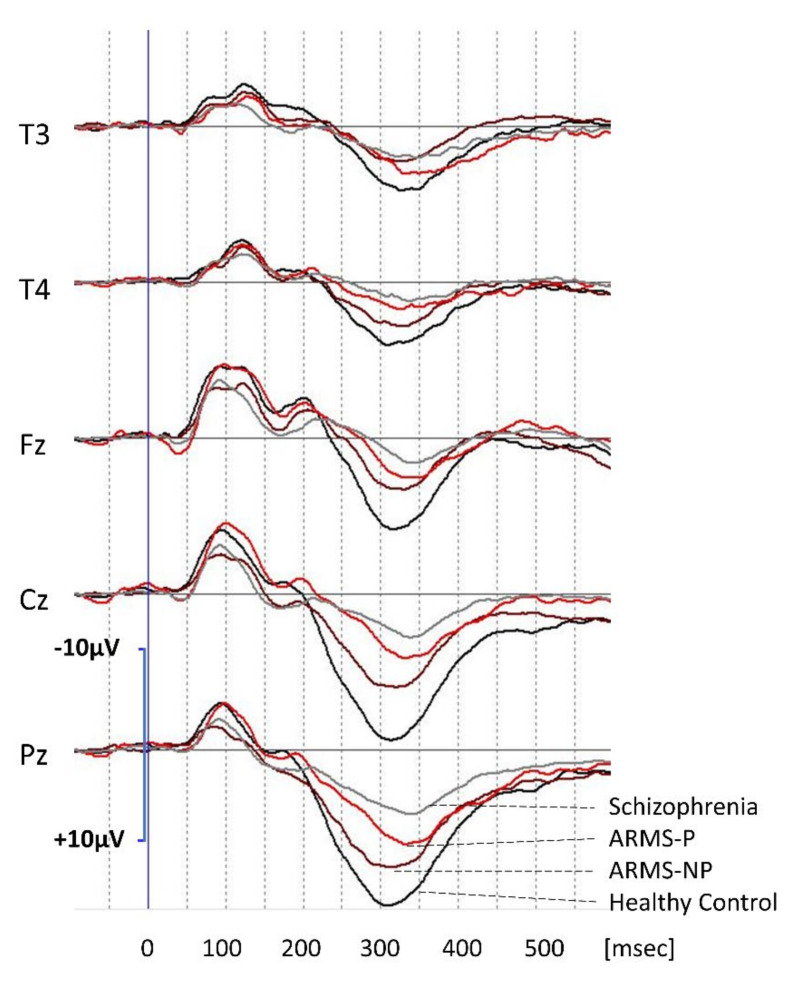
P300 waveforms at the T3, T4, Fz, Cz, and Pz leads. Grand average waveforms for subjects with ARMS who later developed psychosis (ARMS-P; red lines) and those who did not (ARMS non-converters) (ARMS-NP; brown lines). For comparison, waveforms of healthy controls (black lines) and those of patients with schizophrenia (gray lines) are also shown.

Grand average waveforms of P300 in ARMS-P and ARMS-NP are shown in Figure 2. For comparison, waveforms of schizophrenia and controls are also drawn. P300 amplitudes appeared to be most profoundly reduced in patient with schizophrenia, followed by ARMS-P and ARMS-NP, in comparison with healthy controls. However, the difference in amplitude between ARMS-P and ARMS-NP was not significant.

P300 latencies of patients with schizophrenia and ARMS-P appeared to be longer than those of ARMS-NP and healthy controls. Statistical analysis revealed that ARMS-P subjects elicited significantly more prolonged latencies at the T3, Cz, and Pz leads compared with those of ARMS-NP subjects (Table 2).

### 3.4. Relationship between P300 and Cognitive Functions

We examined correlations between P300 parameters and cognitive functions. As shown in Table 3, in entire (ARMS, schizophrenia, and healthy controls combined) subjects, P300 amplitudes at Pz were significantly positively correlated with mGAF scores, performance on some BACS domains (motor function, attention, and executive function) and BACS composite score. Furthermore, P300 latencies were negatively correlated with composite and attention scores of the BACS. In subjects with ARMS as a whole, scores on the mGAF were positively correlated with P300 amplitudes. Such relationship was found in ARMS-NP (r_s_ = 0.57, *p* = 0.019) but not in ARMS-P subgroups (data not shown). No significant relationships were observed between performance on the BACS and P300 latency in ARMS subjects. In subjects with schizophrenia, BACS composite scores and PANSS general psychopathology mildly correlated with P300 amplitude, but no relationships were observed between latency and other clinical/cognitive measurements.

## 4. Discussion

To our knowledge, this is the first report that subjects with ARMS who later developed overt schizophrenia (ARMS-P) elicited prolonged P300 latencies at baseline com-pared to those in subjects who did not develop psychosis. Progression to schizophrenia was also associated with disturbances of cognition and social functioning at baseline, as represented by poorer performances on the BACS in ARMS-P subjects compared to those of ARMS-NP. We further demonstrated a significant correlation between P300 amplitudes and global functioning in ARMS subjects.

As shown on Table 1 and Table 2, attention deficits in ARMS-P, but not ARMS-NP subjects, as measured by the BACS, were greater than −1 (z-score), which was comparable to those in overt schizophrenia. Consistent with previous studies [17,19], our results indicated that P300 amplitudes and latencies in the entire patients were significantly associated with performance on the digit symbol substitution test in the BACS (Table 3). These findings accord with the notion that P300 reflects attention-dependent information processing and provides a feasible marker of neurophysiological abnormalities shared by different clinical stages of schizophrenia [17,18,19,20,21,54]. P300 and other EEG parameters have been shown to be influenced by several factors, such as volition and medication status the latter including antipsychotic drugs [9,39,40,55]. Although some previous studies of P300 used subjects with ARMS receiving these drugs [34,35,56], all of the participants studied here were antipsychotic-free, which is one of the strengths of our study.

P300 parameters have been investigated in the context of prediction of people particularly vulnerable to developing psychosis [32,33,34,35,36,38,56,57]. Reduced P300 amplitudes in subjects with ARMS as a whole, studied here, compared to those in healthy controls is consistent with previous findings [32,33,34,35,36,38,56,57]. By contrast, the lack of difference in P300 amplitudes between ARMS-P and ARMS-NP differs from the findings in some previous studies [34,36]. Importantly, no study has reported prolonged latency in ARMS-P subjects compared to ARMS-NP subjects. Part of these discrepant results may be related to the difference in study conditions and/or backgrounds of subjects; one study [34] used longer (100 ms) duration for the target tone, unlike the present study used 50 ms duration of target tone. Further, other studies [34,35,56] used patients who were treated with antipsychotic drugs, while our study concerned only antipsychotic-free patients. The discrepancy in presence/absence of prolonged P300 latency between a previous study [33] that also used antipsychotic-free patients and the current one may be explained by the difference in demographic data, such as age, a factor known to prolong P300 latency [58,59].

The origins of the P300 are considered to involve the temporal-parietal junction, medial temporal cortex, and lateral prefrontal cortex [60,61]. For example, volume reductions of these brain regions have been found to be correlated with decreased P300 amplitudes in ARMS subjects [35,56]. Further, the supramarginal gyrus, a part of temporal-parietal junction whose function is impaired in schizophrenia [62], has been regarded as one of the main generators of P300, because its damage results in deterioration of P300 activity [63]. Thus, the aberrant functions of these brain areas, such as temporal gyrus, may cause prolongation of P300 latency in schizophrenia and ARMS. Further, re-ductions in P300 amplitudes and prolonged latency are also present in unaffected relatives of patients [64,65,66,67], suggesting genetic backgrounds for these neurophysiological phenotypes. Specifically, data from a meta-analysis performed by Bramon et al. (2005) indicate a significant prolongation of P300 latency in relatives of schizophrenia patients, while its amplitudes were not affected [64]. Further efforts to characterize these electro-physiological manifestations in the prodromal stage of schizophrenia are likely to broaden the views on trait markers of the illness.

Cognitive and social functioning has been recognized to provide an important target of early intervention for people susceptible to developing psychosis. The SCoRS was developed for measuring daily activity skills in patients with schizophrenia [49]. Further, we found this assessment tool is also useful for evaluation of real-world activities in people with ARMS [47]. Importantly, SCoRS scores in the ARMS-P group were worse than those in ARMS-NP group (Table 2). To our knowledge, our observation is the first to indicate that daily-living skills linked to cognition, evaluated in this way, are more profoundly impaired who later convert to schizophrenia compared to non-converters. These findings overall add to the concept that poor functionality provide a trait marker for psychosis.

There have been attempts to identify neural substrates for neuropsychological disturbances of individuals in early stages of schizophrenia. Accordingly, P300 indices at Pz elicited significant correlations with mGAF and BACS scores in entire subjects (healthy controls, ARMS subjects and patients with schizophrenia combined) (Table 3). These findings are consistent with the concept that changes of cognitive and social functions, measured behaviorally, are reflected by some electrophysiological parameters in individuals with schizophrenia or the clinical high-risk state [9,38,39]. On the other hand, P300 amplitudes were related to mGAF, but not BACS scores in the ARMS subjects. Further study with a larger number of subjects should clarify the contribution of P300 activities to functional outcomes in ARMS.

Finally, it would be worthwhile to discuss the clinical implications of our findings. The present results indicate prolonged P300 latency may provide a potential biomarker for schizophrenia. This is supported by the lack of a significant effect of antipsychotic treatments on P300 latency, unlike the case for its amplitudes, as demonstrated in a meta-analysis [68], suggesting the robustness of P300 latency as a trait-marker of schizophrenia. On the other hand, a number of other candidates for biomarkers of the disease have been reported, and attempts have been made to combine multiple indicators to improve predictive accuracy [69,70]. In this line, P300 latency may help improve diagnostic accuracy when used with other indices.

Several limitations of this study should be considered. First, the small sample size, especially for ARMS-P subjects, might have limited the statistical power and restricted the generalizability of our results. Second, given the evidence of age-related changes of P300 [59,71], the higher age of schizophrenia patients as compared with other groups might have biased our results. However, this may not be relevant to the difference in P300 laten-cy between the ARMS-P and -NP groups (the main find of the current study), as they shared similar ages.

In conclusion, the present study suggests, for the first time, the ability of P300 latency to predict the development of schizophrenia in vulnerable subjects. Our observations also confirm that poor cognitive function and daily-living skills are associated with the risk of development of schizophrenia. When combined with other indices of different modalities, P300 latency may provide a diagnostic marker. Further study is warranted to investigate if P300 latency would be linked to outcomes and other clinical features of individuals at high-risk for schizophrenia and related psychoses.

## Figures and Tables

**Table 1:** Demographic data, cognitive functions and P300 parameters

	H	ARMS	Sch	Effect Size	Group Comparison
	(*n* = 28)	(*n* = 33)	(*n* = 39)		
Male/female	16/12	23/10	16/23	-	χ^2^ = 6.00, *p* = 0.05, n.s.
Age (years)	21.7 (5.0)	19.2 (4.6)	24.4 (7.2)	-	F(2,97) = 7.93, *p* = 0.001 **, ARMS < Sch
Age of onset (years)	-	-	24.4 (6.6)	-	-
Duration of illness (years)	-	-	2.7 (3.0)	-	-
JART ^a^	102.5 (7.4)	96.9 (10.0)	99.3 (11.1)	-	F(2,85) = 1.91, *p* = 0.15, n.s.
PANSS: positive symptom	-	13.1 (3.7)	16.7 (5.8)	-	*p* = 0.003 **, ARMS < Sch
negative symptom	-	19.5 (7.2)	19.1 (7.4)	-	*p* = 0.81, n.s.
general psychopathology	-	34.9 (8.9)	36.5 (9.1)	-	*p* = 0.45, n.s.
mGAF	-	46.5 (9.1)	34.1 (8.6)	-	*p* < 0.001 **, ARMS > Sch
SCoRS	-	4.7 (2.2)	5.8 (2.3)	-	*p* = 0.069, n.s.
BACS ^a,b^: verbal memory	−0.16 (1.1)	−0.54 (1.4)	−1.46 (1.6)	0.12	F(2,86) = 5.81, *p* = 0.004 **, H, ARMS > Sch
working memory	0.10 (0.8)	−0.9 (1.3)	−1.32 (1.3)	0.15	F(2,86) = 7.99, *p* = 0.001 **, H > ARMS, Sch
motor function	−0.15 (1.0)	−1.31 (1.7)	−2.04 (1.5)	0.18	F(2,86) = 9.45, *p* < 0.001 **, H > ARMS, Sch
verbal fluency	0.096 (1.0)	−0.96 (1.2)	−1.44 (1.3)	0.19	F(2,86) = 9.84, *p* < 0.001 **, H > ARMS, Sch
attention	0.70 (0.8)	−0.22 (1.5)	−1.60 (1.3)	0.32	F(2,86) = 20.97, *p* < 0.001 **, H > ARMS > Sch
executive function	0.29 (1.1)	−0.36 (1.3)	−1.47 (1.9)	0.16	F(2,86) = 8.64, *p* < 0.001 **, H, ARMS > Sch
composite score ^c^	0.14 (0.5)	−0.72 (1.0)	−1.55 (1.2)	0.28	F(2,86) = 17.52, *p* < 0.001 **, H > ARMS > Sch
P300 Amplitude (µV):T3	9.26 (4.5)	6.12 (3.1)	5.14 (2.6)	0.20	F(2,97) = 11.76, *p* < 0.001 **, H > ARMS, Sch
T4	9.30 (5.5)	6.50 (4.1)	4.04 (3.0)	0.20	F(2.97) = 12.19, *p* < 0.001 **, H > ARMS, Sch
Fz	13.59 (7.2)	8.46 (6.3)	4.80 (4.2)	0.27	F(2,97) = 17.89, *p* < 0.001 **, H > ARMS > Sch
Cz	19.51 (8.6)	13.08 (6.3)	7.07 (3.6)	0.40	F(2,97) = 32.33, *p* < 0.001 **, H > ARMS > Sch
Pz	20.57 (8.9)	15.66 (4.9)	9.4 (3.9)	0.37	F(2,97) = 28.39 *p* < 0.001 **, H > ARMS > Sch
P300 Latency (msec):T3	327.8 (35.9)	325.5 (32.5)	347.1 (52.9)	0.07	F(2,97) = 2.78, *p* = 0.067, n.s.
T4	326.3 (29.5)	320.1 (39.0)	343.4 (52.6)	0.06	F(2,97) = 2.88, 0.061, n.s.
Fz	323.6 (30.9)	322.9 (38.6)	343.9 (52.7)	0.05	F(2,97) = 2.76, *p* = 0.068, n.s.
Cz	314.8 (29.1)	315.3 (36.1)	340.5 (52.2)	0.08	F(2,97) = 4.43, *p* = 0.014 *, H, ARMS < Sch
Pz	313.5 (29.5)	314.9 (32.5)	339.0 (45.7)	0.10	F(2,97) = 5.17, *p* = 0.007 **, H, ARMS < Sch

Values represent mean (SD). ARMS; at-risk mental state, H; healthy controls, Sch; schizophrenia. JART; Japanese Adult Reading Test, PANSS; Positive and Negative Syndrome Scale, mGAF; modified Global Assessment Functioning. P300 data represent peak amplitudes (µV) and latencies (msec) for each group [mean (SD)]. All participants were antipsychotic-free at the time of measurement P300. Demographic differences between groups were examined with one-way analysis of variance (ANOVA), Student’s *t*-test or the chi-square test (* *p* < 0.05, ** *p* < 0.01). Effect sizes are represented in η^2^. ^a^ BACS and JART data were missing for some healthy control patients. ^b^ BACS was standardized by creating z-scores, with the mean score of Japanese healthy controls set to zero and the standard deviation set to one [45]. ^c^ BACS composite score was calculated by averaging all z-scores of the six primary measures from the BACS [46].

**Table 2:** Demographic data, cognitive functions and P300 parameters in clinical high-risk subjects.

	ARMS-NP (*n* = 25)	ARMS-P (*n* = 8)	Group Comparison
Male/female	17/8	6/2	χ^2^ = 0.14, *p* = 0.70, n.s.
Age (years)	18.7 (3.8)	20.5 (6.6)	*p* = 0.33, n.s.
JART	98.1 (9.6)	93.3 (11.1)	*p* = 0.25, n.s.
PANSS: positive symptom	12.9 (4.1)	13.8 (2.1)	*p* = 0.53, n.s.
negative symptom	19.6 (8.0)	19.2 (4.3)	*p* = 0.85, n.s.
general psychopathology	34.1 (9.1)	37.2 (8.4)	*p* = 0.40, n.s.
mGAF	48.6 (9.0)	41.7 (7.9)	*p* = 0.096, n.s.
SCoRS	4.1 (1.9)	6.6 (2.0)	*p* = 0.004 **
BACS: verbal memory	−0.36 (1.4)	−1.1 (1.3)	*p* = 0.21, n.s.
working memory	−0.65 (1.1)	−1.70 (1.8)	*p* = 0.060, n.s.
motor function	−1.09 (1.8)	−2.02 (1.4)	*p* = 0.20, n.s.
verbal fluency	−0.78 (1.1)	−1.50 (1.4)	*p* = 0.16, n.s.
attention	0.06 (1.5)	−1.12 (1.4)	*p* = 0.063, n.s.
executive function	−0.17 (0.96)	−0.94 (2.2)	*p* = 0.17, n.s.
composite score	−0.50 (0.94)	−1.40 (1.25)	*p* = 0.038 *
Amplitude (µV):T3	6.06 (3.0)	6.30 (3.8)	*p* = 0.85, n.s.
T4	7.18 (3.9)	4.37 (4.4)	*p* = 0.099, n.s.
Fz	8.94 (6.6)	6.96 (5.7)	*p* = 0.45, n.s.
Cz	14.14 (6.0)	9.79 (6.7)	*p* = 0.094, n.s.
Pz	16.27 (5.2)	13.76 (3.3)	*p* = 0.21, n.s.
Latency (msec):T3	318.9 (30.5)	346.5 (31.8)	*p* = 0.039 *
T4	317.2 (40.0)	329.0 (36.6)	*p* = 0.49, n.s.
Fz	315.9 (35.7)	344.7 (41.3)	*p* = 0.65, n.s.
Cz	306.5 (34.5)	342.7 (27.3)	*p* = 0.011 *
Pz	307.7 (29.3)	337.5 (33.3)	*p* = 0.022 *

Values represent mean (SD). ARMS; at-risk mental state, ARMS-*p*; ARMS patients later transitioned to overt psychosis, ARMS-NP; ARMS patients who did not transition to overt psychosis, JART; Japanese Adult Reading Test, PANSS; Positive and Negative Syndrome Scale, mGAF; modified Global Assessment Functioning, SCoRS; Schizophrenia Cognitive Rating Scale, BACS; Brief Assessment of Cognition in Schizophrenia. P300 data represent peak amplitudes (µV) and latencies (msec) for each group [mean (SD)]. Demographic differences between groups were examined with Student’s *t*-test or the chi-square test (* *p* < 0.05, ** *p* < 0.01).

**Table 3:** Correlations between P300 parameters and clinical symptoms.

	Entire Subject ^a^				ARMS (*n* = 33)				Schizophrenia (*n* = 39)			
	Amplitude (µV)		Latency (msec)		Amplitude (µV)		Latency (msec)		Amplitude (µV)		Latency (msec)	
	r_s_	*p*	r_s_	*p*	r_s_	*p*	r_s_	*p*	r_s_	*p*	r_s_	*p*
JART	−0.03	0.77	0.08	0.44	−0.08	0.65	0.07	0.71	0.10	0.56	0.01	0.95
PANSS: positive symptom	−0.15	0.20	0.02	0.86	−0.08	0.68	0.05	0.78	0.12	0.47	−0.11	0.50
negative symptom	−0.19	0.12	0.21	0.09	−0.30	0.09	0.15	0.41	−0.28	0.10	0.31	0.06
general psychopathology	−0.22	0.07	0.08	0.51	−0.08	0.68	0.17	0.34	−0.34	0.04 *	−0.022	0.90
mGAF	0.46	0.003 **	−0.14	0.29	0.48	0.02 *	0.09	0.67	0.14	0.46	−0.095	0.61
SCoRS	−0.22	0.07	0.17	0.16	−0.11	0.54	0.16	0.36	−0.17	0.33	0.11	0.52
BACS: verbal memory	0.25	0.01	−0.10	0.35	0.02	0.65	−0.02	0.92	0.31	0.06	−0.04	0.80
working memory	0.21	0.04	−0.19	0.06	−0.21	0.23	−0.15	0.40	0.28	0.10	−0.12	0.48
motor function	0.41	<0.001 ^b^	−0.22	0.04	0.03	0.86	−0.28	0.10	0.27	0.10	−0.14	0.54
verbal fluency	0.28	0.007	−0.24	0.02	−0.03	0.87	−0.17	0.34	0.17	0.31	−0.85	0.62
attention	0.50	<0.001 ^b^	−0.33	0.001 ^b^	0.06	0.74	−0.16	0.36	0.43	0.01	−0.24	0.14
executive function	0.36	0.003 ^b^	−0.15	0.14	0.06	0.74	−0.002	0.99	0.37	0.03	−0.20	0.22
BACS composite score	0.43	<0.001 **	−0.28	0.006 *	0.01	0.95	−0.22	0.20	0.34	0.04 *	−0.16	0.33

Data were calculated by Spearman’s rank correlation (* *p* < 0.05, ** *p* < 0.01). ^a^ “Entire subject” means schizophrenia, ARMS, and healthy control (*n* = 100). As shown in Table 1, PANSS, mGAF and SCoRS were measured only in ARMS and schizophrenia patients (*n* = 72). ^b^ BACS subdomains survived after Bonferroni’s correction for multiple comparison.

## Data Availability

The data presented in this study are available on request from the corresponding author. The data are not publicly available since we do not have permission to share the data.
